# Non-neuronal cholinergic system in the heart influences its homeostasis and an extra-cardiac site, the blood-brain barrier

**DOI:** 10.3389/fcvm.2024.1384637

**Published:** 2024-03-27

**Authors:** Yoshihiko Kakinuma

**Affiliations:** Department of Bioregulatory Science, Graduate School of Medicine, Nippon Medical School, Tokyo, Japan

**Keywords:** non-neuronal cholinergic system, non-neuronal cardiac cholinergic system, acetylcholine, blood brain barrier, heart, brain

## Abstract

The non-neuronal cholinergic system of the cardiovascular system has recently gained attention because of its origin. The final product of this system is acetylcholine (ACh) not derived from the parasympathetic nervous system but from cardiomyocytes, endothelial cells, and immune cells. Accordingly, it is defined as an ACh synthesis system by non-neuronal cells. This system plays a dispensable role in the heart and cardiomyocytes, which is confirmed by pharmacological and genetic studies using murine models, such as models with the deletion of vesicular ACh transporter gene and modulation of the choline acetyltransferase (ChAT) gene. In these models, this system sustained the physiological function of the heart, prevented the development of cardiac hypertrophy, and negatively regulated the cardiac metabolism and reactive oxygen species production, resulting in sustained cardiac homeostasis. Further, it regulated extra-cardiac organs, as revealed by heart-specific ChAT transgenic (hChAT tg) mice. They showed enhanced functions of the blood-brain barrier (BBB), indicating that the augmented system influences the BBB through the vagus nerve. Therefore, the non-neuronal cardiac cholinergic system indirectly influences brain function. This mini-review summarizes the critical cardiac phenotypes of hChAT tg mice and focuses on the effect of the system on BBB functions. We discuss the possibility that a cholinergic signal or vagus nerve influences the expression of BBB component proteins to consolidate the barrier, leading to the downregulation of inflammatory responses in the brain, and the modulation of cardiac dysfunction-related effects on the brain. This also discusses the possible interventions using the non-neuronal cardiac cholinergic system.

## 1 Introduction

The final product of the non-neuronal cholinergic system of the cardiovascular system is acetylcholine (ACh), which is not derived from the parasympathetic nervous system but from cardiomyocytes and endothelial cells. This system plays a protective and physiologically dispensable role specifically in the heart and cardiomyocytes and the system in the heart is named as the non-neuronal cardiac cholinergic system (NNCCS). This system sustains the physiological function of the heart and prevents the development of cardiac dysfunction. Moreover, the system regulates brain functions via modulating the blood-brain barrier (BBB). Therefore, NNCCS influences the heart and brain because each organ is innervated by the vagus nerve. Although the NNCCS is equipped by the heart, the physiological functions are reported to be more crucial than expected by many studies.

## 2 History of the non-neuronal cardiac cholinergic system (NNCCS)

A pioneering study revealed that cardiomyocytes or myocardium derived from 1-day-old *Xenopus* embryos release acetylcholine (ACh), which is upregulated by acetylcholinesterase inhibitor, and the cells express the immunoreactivity of choline acetyltransferase (ChAT) (1). Another study demonstrated that ChAT activity is highest in the sinoatrial node, followed by the atrioventricular node and conduction bundles. However, the activity was one-third of the conduction system in the right and left ventricles of the guinea pig heart (2). Moreover, a transplanted rat heart without nerve innervation maintained an ACh level in each cardiac ventricle and atrium comparable to that of a control heart (3). Therefore, these previous studies strongly suggest that cardiomyocytes are capable of synthesizing ACh, which differs from the ACh released from the vagus nerve ends.

Since the immunohistochemical study by Fu et al. in 1998 and the study by Oda et al. in 1987 measuring regional ACh contents in the heart, the evidence of the presence of ACh in cardiomyocytes has accumulated. However, the significance and biological roles of cardiomyocyte-derived ACh remained unclear until 2009, when it was demonstrated that cardiomyocyte-derived ACh plays an important role in negatively regulating the overshoot of oxygen consumption, probably through mitochondria (4). This study revealed the biological significance of the system by establishing the link between the cardiomyocytes-derived ACh synthesis system and their energy metabolism. Simultaneously, another study by Rana et al. revealed that ACh is synthesized in the heart, both in the atrium and ventricle, and that ACh levels decrease in an age-dependent manner (5), indicating that this system is influenced by aging.

Various studies have established that cardiomyocytes possess the machinery to synthesize ACh through a system different from the parasympathetic nervous system (PNS), such as the vagus nerve. Therefore, ACh is derived from the PNS and ACh synthesis system in the heart. The latter system has been referred to as the non-neuronal ACh in the heart, also referred to as the non-neuronal cholinergic system in the heart, the cardiac non-neuronal cholinergic system, or non-neuronal cardiac cholinergic system (NNCCS). In our review, NNCCS is mainly used.

Prado et al. investigated the significance of this system using the acetylcholinesterase inhibitor pyridostigmine in rat cardiomyocytes. Their results indicated the treated cardiomyocytes increased nitric oxide (NO) production and suppressed isoproterenol-induced hypertrophy as well as the alteration of calcium responses induced by adrenergic overstimulation (6). Further, cardiomyocytes from knockout mice lacking the vesicular ACh transporter (VAChT) gene (VAChT KO mice) had disturbed cardiac activity, including exaggerated exercise-induced elevation of heart rate, poor recovery of heart rate after exercise, increased exposure to mitochondrial reactive oxygen species (ROS) stress, and increased peak calcium transients (7). Notably, these dysregulated cardiac functions contributed to cardiac hypertrophy and disturbed responses to a β-stimulant, isoproterenol (7). These characteristics of VAChT KO mice revealed that cardiomyocyte-secreted ACh is indispensable for sustaining the physiological homeostasis of the heart.

In 2013, two different types of mice−hChAT tg (8) and VAChT KO (7)—were studied, and their effects on the heart were compared. hChAT tg mice overexpressing the ChAT gene showed enhanced NNCCS function because of extremely high cardiac ACh levels (8). Cardiomyocytes from hChAT tg mice were less dependent on oxygen, and the hearts from hChAT tg mice were able to utilize glucose more efficiently as a fuel source than wild-type (WT) mice, as more GLUT4 was expressed in the hearts of hChAT tg mice. Therefore, hChAT tg hearts were more resilient to hypoxia, ischemia, or myocardial infarction (MI) due to enhanced protein expression levels of hypoxia-inducible factor (HIF)-1α, even under normoxic conditions. The MI-subjected hearts of ChAT tg mice contained more new capillaries and intact cardiomyocytes. Despite comparable hemodynamic and cardiac parameters, the ChAT tg mouse heart, which was connected to a Langendorf apparatus, continued to beat for a more prolonged period, even after the perfusion had stopped. Further, the heartbeat started more rapidly than in the WT mouse heart after reperfusion. Consequently, we attempted to explore and consider the critical biological role of the NNCCS. For example, the NNCCS regulates cardiac energy metabolism, and the augmentation of this system increases glucose uptake as a fuel substrate and enhances angiogenesis, resulting in enhanced resistance to hypoxia and ischemia in the heart. The same research group developed a different murine NNCCS dysfunction model (9) from VAChT KO and heart-specific ChAT KO mice (7). Heart-specific ChAT knockdown (hChAT KD) mice, developed by simultaneously overexpressing three ChAT mRNA-specific microRNAs in the heart, showed typical cardiac dysfunction with enhanced expression of heart failure markers, dysregulated expression of glucose metabolism-related molecules, increased ROS exposure in the heart, impaired cardiac NO production, poor angiogenesis, blunted vagus nerve activity, and disturbed association between NO synthase 1 and SERCA2 protein. Therefore, hChAT KD mice shared the phenotypes induced by impaired NNCCS functions with VAChT KO mice.

In addition, Roy et al. extensively examined the influence on cardiac function in angiotensin II-treated VAChT KO mice and heart-specific ChAT KO mice using the Cre-loxP system (10) and revealed that either KO mice comparably showed impaired cardiac functions, especially when challenged by hemodynamic stress. However, the precise underlying mechanisms and targets by which cardiac function is impaired remain unclear.

## 3 Summary of a critical function of the NNCCS in the heart

The following section summarizes the results of various previous studies that focused on the physiological functions of the NNCCS.

First, the NNCCS protects the heart from various stressors such as hypoxia, ischemia, ROS, and inotropic agents (6–8, 10, 11). Notably, the induction of HIF-1α protein through the non-hypoxic pathway is responsible for this resilience (12). HIF-1α, a crucial transcription factor for an anti-hypoxic or anti-ischemic response, shifts cellular energy metabolism predominantly to glucose metabolism.

Second, the NNCCS plays an inhibitory role in conditions with the enhanced sympathetic nervous system where cardiac function is activated for compensation. The NNCCS also attenuates cardiac hypertrophy or cardiac remodeling and efficiently decreases heart rate in response to stimuli (6–8, 10, 11).

Third, the NNCCS positively regulates cell−cell interactions in the heart by sustaining gap junction function by stabilizing connexin and β-catenin localization in cardiomyocytes. Downregulation of the NNCCS causes mislocalization of connexin and β-catenin from the plasma membrane of cardiomyocytes, leading to decreased immunoreactivity (11). The localization of gap junction proteins at the proper site in a cardiomyocyte indicates that the NNCCS plays a role in sustaining electrophysiological properties in the heart and inhibits the genesis of an arrhythmogenic reentry circuit.

Fourth, the NNCCS regulates angiogenesis in the heart, which is closely related to heart protection. Without the NNCCS, angiogenesis impairment may blunt compensatory adaptation of the heart and aggravate cardiac functions, especially in a post-myocardial infarction state (8).

These four characteristics are novel physiological findings related to the role of NNCCS in the heart. Among the previous studies dealing with cardiogenic factors synthesized by cardiomyocytes, ACh, to the best of my knowledge, may be one of the factors exerting pleiotropic effects on the heart, independent of its already known negative inotropy and chronotropic effects.

## 4 NNCCS remotely influences brain function

Additionally, based on previous studies using hChAT tg mice (13, 14), the NNCCS plays a significant role in regulating higher brain functions, including mood or stress (13), and the blood-brain barrier (BBB) (14). hChAT tg mice showed anti-stress and anti-depressive phenotypes because the immobility duration was more shortened in the tail suspension and forced swimming tests than in WT mice. Further, the peak corticosterone levels in hChAT tg mice during restraint stress were also lower than those in WT mice, and hChAT tg mice stayed for a longer time in the open space of the elevated plus maze. In addition, they were more resistant to convulsant inducers and less susceptible to status epileptics that causes death. Furthermore, these specific central nervous system (CNS) phenotypes were dependent on the vagus nerve because the vagus nerve activity of hChAT tg mice was significantly enhanced, and the phenotypes almost overlapped with those of vagus nerve-stimulated WT mice. However, left vagotomy or treatment with L-NAME, an NOS inhibitor, clearly reversed almost all specific phenotypes of hChAT tg mice (13). Therefore, CNS functions in hChAT tg mice may be modulated by enhancing vagus nerve activity and increasing cardiac NO production. Alternatively, hChAT tg mice exhibited enhanced vagus nerve activity like a mouse subjected to the intrinsic vagus nerve stimulation (VNS).

As one of the clues as to the CNS phenotypes of ChAT tg mice, claudin-5, a BBB protein, was significantly expressed in their brains compared with WT mice, and the function of BBB was more consolidated under both physiological and pathological conditions (14). Under physiological conditions, without any manipulation of ChAT tg mice, basal claudin-5 protein expression levels were significantly elevated in the whole brain, whose immunoreactive signals were evident in the hippocampus. Therefore, the ChAT tg mouse brain significantly attenuated Evans blue leakage in a cold injury model, which is a well-known method to evaluate BBB integrity. Furthermore, in the 1-methyl-4-phenyl-1, 2, 3, 6-tetrahydropyridine (MPTP)-induced Parkinson's disease murine model, the disruption of BBB was significantly attenuated in hChAT tg mice, and MPTP-induced neuronal loss in the substantia nigra was significantly suppressed. This indicates that consolidated BBB function via claudin-5 is a key molecular phenotype in augmented NNCCS. However, the link between the claudin-5 protein and the NNCCS or precise mechanism by which VNS regulates BBB function remains to be fully elucidated.

## 5 Link between the vagus nerve and BBB

VNS exerts beneficial effects on remote organs innervated by the vagus nerves. Notably, VNS exerts anti-inflammatory (suppression of cytokine levels and inflammasome formation) and anti-apoptotic effects. An α7 nicotinic receptor stimulated by ACh is responsible for anti-inflammation. Here, we would like to discuss results from several animal studies using VNS and speculate the underlying mechanisms for VNS effects on BBB from several VNS aspects.

First, VNS negatively regulates the progression of inflammation in the brain. This cholinergic anti-inflammatory pathway, represented by ACh released from VNS, was initially reported by Borovikova et al. in an LPS-induced endotoxemia model (15). Thereafter, in brain injury models, VNS has been reported to exert anti-inflammatory responses, including a reduction of local responses or systemic levels of pro-inflammatory cytokines. Conversely, VNS also enhanced anti-inflammatory cytokines, including IL-10 (16–18).

Second, VNS upregulates the norepinephrine system in the brain. Upregulation of the norepinephrine levels in the brain leads to functional improvement in animals post-brain injury. In contrast, blockade of the norepinephrine pathway, including lesions of the locus coeruleus (LC), depletes norepinephrine levels in the brain, leading to poor brain functional recovery post-injury (19–22).

Third, VNS decreases brain edema, probably through the modulation of aquaporin (AQP)-4 (23). In a rat model of unilateral fluid percussion brain injury, VNS improved brain injury-induced locomotor dysfunction, probably by reducing the injury-related brain edema that appeared following injury (24). Brain injury-induced brain edema is a post-injury event that increases intracerebral pressure, compromising brain perfusion. Therefore, the suppressive effects of VNS on brain edema are promising. Edema occurs because of increased interstitial fluid storage in the brain vasculature through AQP-4, an intracellular water transport channel. As reported by Lopez, the down-regulation of AQP-4 immunoreactivity in the perivascular regions of the injured brain is one of the underlying mechanisms for VNS-induced attenuation of brain edema (23).

Fourth, several animal and preclinical studies have reported that VNS influences BBB function to prevent the disruption of BBB in a pathological model (25–27). This effect of VNS is closely linked to the reduction of edema, as the reduced BBB function directly causes brain edema. Moreover, VNS-induced attenuation of proinflammatory cytokines also conserves BBB, as these cytokines produced in the brain inhibit expression of tight junction components, including claudin-5 and occludin. Therefore, BBB function is protected by VNS. Notably, VNS also attenuated intestinal permeability regulated by tight junctions in the epithelia in a brain injury model as well as in other inflammatory models (28, 29). The ends of the vagus nerve are not only distributed in the gastrointestinal system as a peripheral organ but also to the brain as a central organ, both of which are influenced by VNS as the vagus nerve includes afferent and efferent fibers. Therefore, the effects of VNS on tight junctions in the BBB or intestine may be mediated by similar mechanisms.

**Figure 1:**
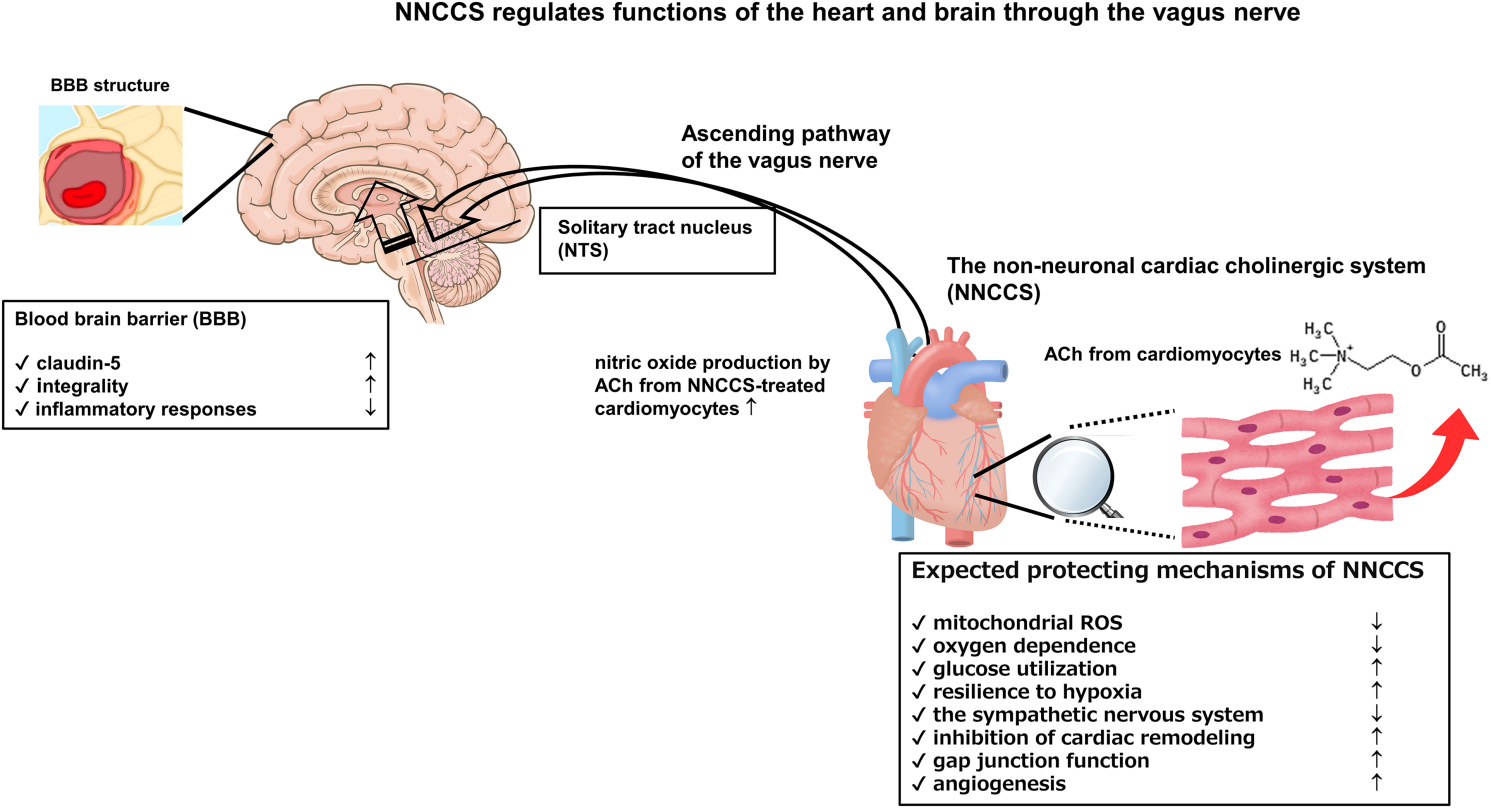
Activation of NNCCS can regulate functions of the heart and brain because the ascending pathway of the vagus nerve transduces the enhanced signal from the NNCCS to the brain.

Moreover, an α7 nicotinic receptor agonist, PNU-282987, strengthened the evidence that the VNS modality on BBB function is mediated by α7 nicotinic receptor-mediated signaling (30). Further, PNU-120596, a selective type-II positive allosteric modulator (α7-PAM) of α7 nicotinic receptor, protected the rat brain from traumatic brain injury (31). Moreover, Kimura et al. using rat brain endothelial cells reported that an α7 nicotinic receptor agonist, PHA543613, upregulated the protein expression of claudin-5 and occludin, strengthening BBB function (32). However, several other studies have reported the opposite conclusion regarding an α7 nicotinic receptor; therefore, it remains controversial (33). The Figure shows crucial aspects of the NNCCS in terms of its action mechanisms and relationship between the NNCCS and BBB Figure 1.

## 6 Pathway connecting the vagus nerve to the vasculature in the brain

These aforementioned studies strongly suggest that the VNS, or vagus nerve, regulates BBB function. However, the mechanisms by which VNS transduces its signals directly or indirectly to the BBB remain unresolved. First, the anatomical pathway, through which the nerve ends projecting from the main nucleus organizing visceral information connect to the brain vasculature, remains under-studied. Second, which receptor−muscarinic or adrenergic−on brain endothelial cells are directly stimulated, remains unknown. Further, mechanisms that indirectly influence the BBB via VNS also remain unexplored.

The predominant afferent pathway of the vagus nerve transduces signals from visceral organs to the solitary tract nucleus (NTS). The information received by NTS is partly transferred into the LC (34, 35), from which the information is propagated through the noradrenergic pathway into the whole brain. Moreover, information from the NTS is projected to other regions, including the hypothalamus, especially the paraventricular hypothalamic nucleus (PVN) (36, 37), the dorsal nucleus of the vagus nerve (38), and the forebrain (39) or telencephalon (40). Although projection pathways from the NTS or LC have been examined using antero- or retro-grade tracers, the precise anatomical or histological evidence remains lacking. Nevertheless, brain vasculature or capillaries possessing BBB properties are influenced or modulated by noradrenergic or cholinergic neurons, as the nerve end of each nervous system may be distributed near the vasculature to regulate vascular tone through each receptor (41). The next sections discuss neurotransmitter-mediated effects on BBB function.

## 7 Possible mechanisms influencing BBB function through adrenergic receptors

The stimulation of the LC with carbachol, a muscarinic receptor agonist, reduced hemispheric cerebral blood flow and increased vascular permeability in the brain (41). Moreover, another study reported that α-adrenergic receptors stimulated brain permeability, attenuating BBB function, whereas β-adrenergic receptors strengthened BBB function (42). Several other studies have also supported these findings using noradrenaline or its antagonists (43–45). Notably, noradrenergic neurons modulate BBB functions, depending on α- or β-receptors expressed on brain capillaries. Further, an *in vitro* study using human pulmonary endothelial cells reported that β1- and β2- adrenergic receptors are involved in the stabilization of endothelial barrier permeability and influence BBB integrity (46).

The transgenic mice with enhanced NNCCS function showed increased claudin-5 protein expression in the brain, leading to more consolidated BBB function and preventing brain pathology in injury models (14). Accordingly, the consolidation of BBB is dependent on the vagus nerve, as the neuroactivity of the vagus nerve of the mice was activated, the lateral vagotomy attenuated claudin-5 protein expression (14), and c-fos signals in the NTS was increased with increased NE content in the hypothalamus (13), suggesting that the NTS of the mice is activated. However, these typical phenotypes were not compatible with increased brain permeability during LC stimulation (41). Therefore, it may be hypothesized that β-receptors, not α-receptors, in brain capillaries are involved in the upregulation of claudin-5-mediated BBB consolidation in NNCCS-enhanced mice.

## 8 Possible mechanisms influencing BBB function through cholinergic receptors

In addition to α-adrenergic receptor-mediated mechanisms that aggravate BBB permeability, cholinergic neurons may regulate BBB function. Although muscarinic and nicotinic receptors are found in brain capillaries, cholinergic nerves do not terminate on brain capillaries (47, 48), raising a discrepancy between the presence of these receptors and undetectable cholinergic nerve ends in capillaries (49). This missing link may be explained by the fact that a non-neuronal ACh system that exists in the endothelial cells of the vasculatures can synthesize and secrete ACh (50, 51); however, the amount of ACh secreted by endothelial cells is predicted to be low compared with that of systemically administered ACh.

However, in contrast to the reports that α-adrenergic receptors are responsible for brain edema, direct evidence suggesting that ACh or cholinergic receptors, not VNS, play a role in sustaining BBB functions is limited. Notably, one study showed that a systemic infusion of ACh decreased blood pressure and increased BBB permeability in normal rats (52). However, this result using a dose affecting blood pressure does not necessarily exclude the possibility that ACh may upregulate BBB function, as demonstrated in our transgenic mice (14), because the underlying mechanism may be different for bolus-injected ACh and constitutively released ACh from endothelial cells.

Several studies have demonstrated the beneficial effects of nicotine in alleviating acute ischemic stroke-induced BBB damage (53). Further, α7 nicotinic ACh receptor agonists prevented BBB disruption in stroke models (54–56). Another study reported that an α7 nicotinic ACh receptor agonist increased the expression of claudin-5 and occludin in endothelial cells *in vitro* (32), suggesting a possible direct link between nicotinic receptor activation and the upregulation of BBB function. Therefore, beneficial effects of VNS on BBB function *in vivo* probably may be mediated by ACh, a transmitter from the cholinergic system, or the synthesis system in endothelial cells. However, evidence of very few cholinergic nerves innervating the microvasculature in the brain may raises issues about the ACh origin.

Considering the modulation of BBB function by adrenergic and cholinergic nervous systems, β-adrenergic and nicotinic receptors may be suitable candidates for augmenting BBB function. Moreover, BBB function is influenced by the upregulated expression of BBB proteins and the anti-inflammatory actions of the cholinergic system. Therefore, BBB function is integratively regulated by both of the aspects.

## 9 Conclusion

Interestingly, NNCCS activation causes activation of the afferent fibers of the vagus nerve, as observed in the VNS, leading to increased consolidation of BBB functions through the upregulation of claudin-5 protein. This critical and specific result may suggest re-considering the link between BBB function and the cholinergic system, including the NNCCS, endothelial ACh synthesis system, and the classic cholinergic nervous system. Moreover, the above studies provide us with a possible modality to positively regulate BBB function. Interventions in the BBB may suppress the progression of the decline in higher brain functions. Therefore, intervention in those cholinergic systems may help elucidate a precise mechanism and modality to regulate the BBB and higher brain functions.
